# Supplementary material for: Sulfur starvation-induced autophagy in Saccharomyces cerevisiae involves SAM-dependent signaling and transcription activator Met4
Source: Nat Commun. 2024 Aug 13;15:6927. doi: 10.1038/s41467-024-51309-6 (PMC11322535; doi:10.1038/s41467-024-51309-6)
Supplement: Supplementary file 1 — Supplementary Information [file 41467_2024_51309_MOESM1_ESM.pdf]

Supplementary information

Sulfur starvation-induced autophagy in *Saccharomyces cerevisiae* involves SAM-dependent signaling and transcription activator Met4

Magali Prigent[1,2], Hélène Jean-Jacques[1], Delphine Naquin[1], Stéphane Chédin[1], Marie-Hélène Cuif[1,2], Renaud Legouis[1,2] and Laurent Kuras[1]*

[1]Université Paris-Saclay, CEA, CNRS, Université Paris-Saclay, Institute for Integrative Biology of the Cell (I2BC), Gif-sur-Yvette, France; [2]INSERM U1280, 91198, Gif-sur-Yvette, France.

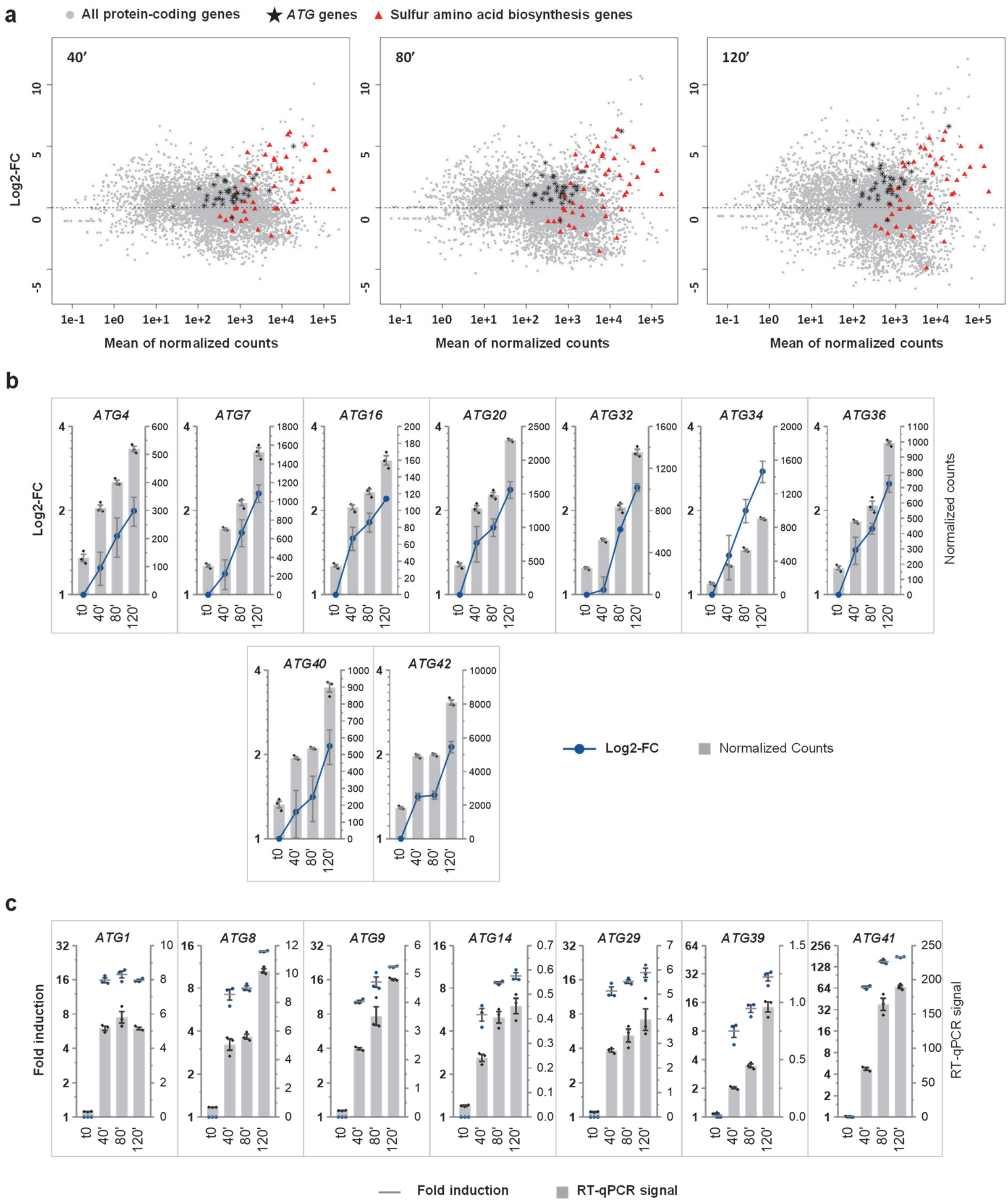

Supplementary Fig. 1. RNA-sequencing analysis of *S. cerevisiae* subjected to sulfur starvation. **a** MA-plot showing log2-FC (y-axis) versus mean of normalized counts (x-axis) for all protein-coding genes (6663 genes). Each point represents a gene. *ATG* genes (black stars) and sulfur amino acid biosynthesis genes (red triangles) are highlighted. **b** Continuation of Fig 1d. Graphs representing log2-FC and normalized counts (main and secondary vertical axes, respectively) at the different time points for the *ATG* genes having a log2-FC between 2 and 3. **c** RT-qPCR on the RNA samples used in the RNA-seq experiment. Induction fold (line, main vertical axis) was calculated relative to t0. mRNA levels (bars, secondary vertical axis) were normalized to 18S ribosomal RNA levels. Data are mean ± SD (dots) or SEM (bars) (n=3 independent experiments). Source data are provided with this paper.

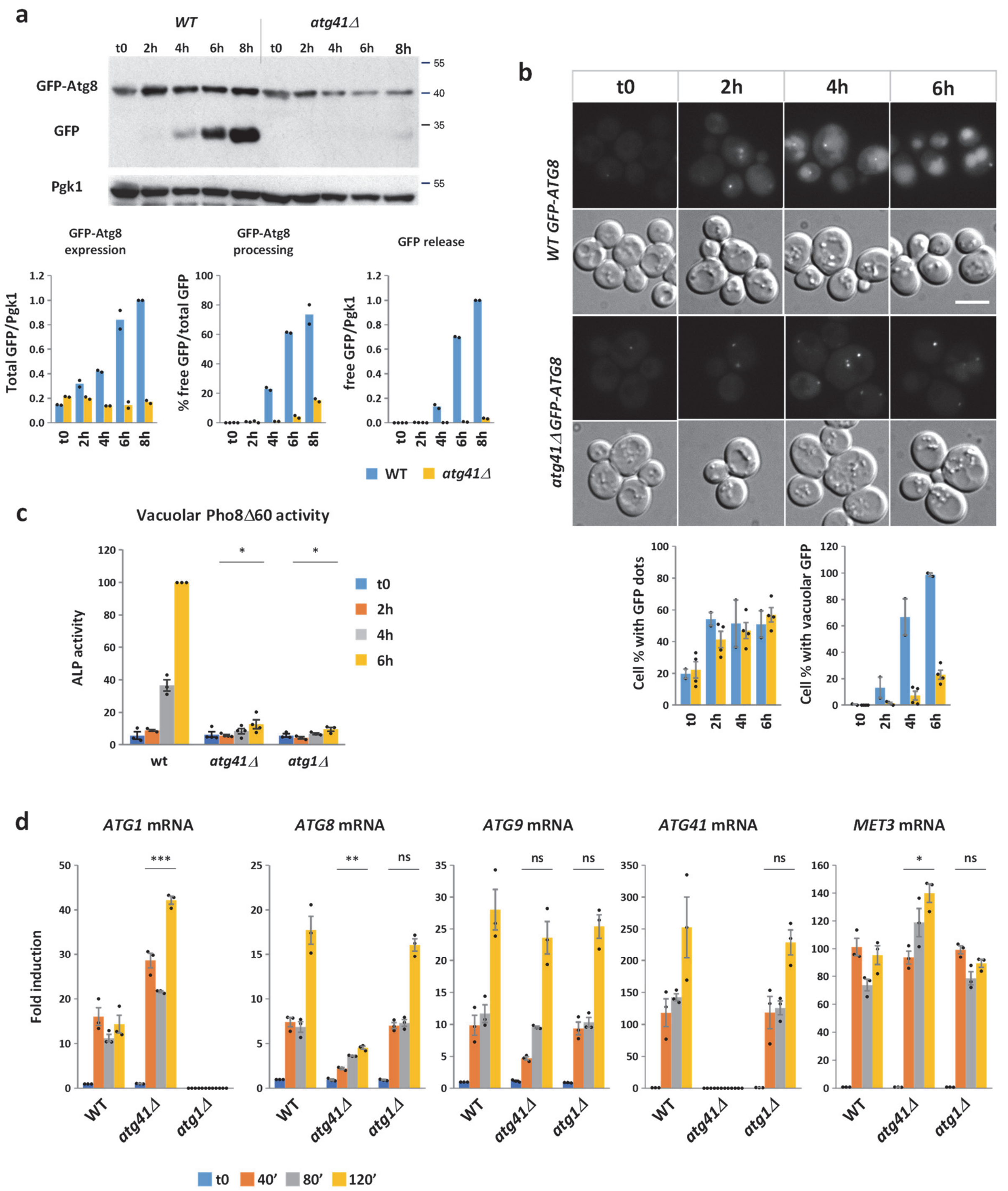

Supplementary Fig. 2. *ATG41* is required for autophagy in response to sulfur depletion. **a** GFP-Atg8 processing assay. WT and *atg41Δ* cells expressing GFP-Atg8 from *ATG8* endogenous promoter (Y1408 & Y1412 or 1413) were subjected to sulfur starvation as in Fig 1. GFP-Atg8 expression and GFP release are relative to the WT at 8h. Data are mean of two independent cultures. **b** Live-cell microscopy. WT and *atg41Δ GFP-ATG8* cells (Y1408 & Y1412 or 1413) were subjected to starvation as above and observed by fluorescence microscopy. Representative images are shown. The graphs indicate the percentage of cells showing GFP dots (left) and accumulating GFP fluorescence in the vacuole (right). Data are mean of two independent experiments using each time two different mutant clones, with in total 200-400 cells scored/ time point. Scale bar, 5 μm. Data for WT GFP-ATG8 in (a) and (b) are the same as in Fig. 1. **c** Pho8Δ60 assay. WT, *atg41Δ* and *atg1Δ* cells expressing pho8Δ60 under the control of *ADH1* promoter (Y1628, Y1643 & Y1638) were subjected to sulfur starvation as above. Vacuolar Pho8Δ60 alkaline phosphatase (ALP) activity was measured as described in *Methods* and is relative to the WT at 6h. Data are mean ± SEM (n=3). Statistical significance compared with WT was determined by two-way ANOVA with Geisser-Greenhouse correction followed by Dunnett's multiple comparisons test. *p = 0.033 and 0.027. **d** RT-

Supplementary Fig. 2 (continued) qPCR analysis. WT, *atg41Δ* and *atg1Δ* cells (BY4742, Y1446 & Y1397) were subjected to sulfur starvation as above. Transcript levels were quantified by RT-qPCR. Fold induction is relative to WT at t0. Data are mean ± SEM (n=3 independent experiments). Statistical significance compared with WT in (c) and (d) was determined as above. P-values, in each case from left to right: ***p < 0.001; **p = 0.0079; *p = 0.019; ns = 0.96, 0.57, 0.91, 0.87 and 0.98. Source data are provided with this paper.

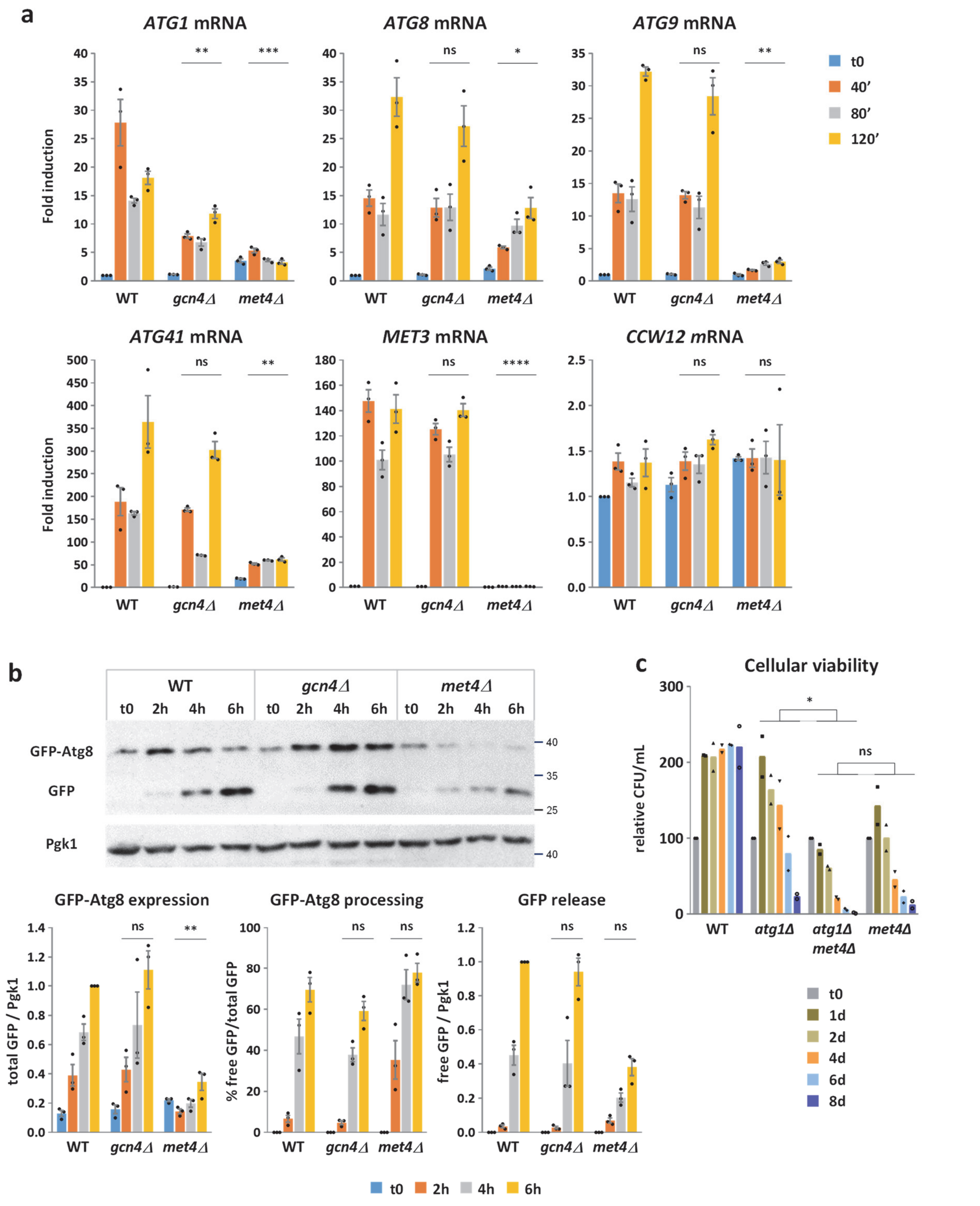

Supplementary Fig. 3. Met4 is essential to induce autophagy upon sulfur starvation in a prototrophic background too. a RT-qPCR analysis. A prototrophic strain and the indicated mutant derivatives (Y1724, Y1746 & Y1786) were grown and subjected to sulfur starvation as in Fig. 4a. Fold induction is relative to WT at t0. Data are mean ± SEM (n=3 independent experiments). Statistical significance with WT was determined by two-way ANOVA with Geisser-Greenhouse correction followed by Dunnett's. P-values: ****p < 0.0001; ***p = 0.0003; **p = 0.0024, 0.0015, 0.0021; *p = 0.036, and ns = 0.88, 0.43, 0.77, 0.19, 0.65 (in each case from left to right). **b** GFP-Atg8 processing assay. A prototrophic strain expressing GFP-Atg8 from *ATG8* endogenous promoter and the indicated mutant derivatives (Y1727, Y1737 & Y1787) were grown and subjected to sulfur starvation as in Fig. 4b. Cells were collected at the indicated times and processed as described in *Methods*. Molecular weight are in kDa. GFP-Atg8 expression and GFP release are relative to the WT at the 6-hour time point. Data are mean ± SEM (n=3 independent experiments). Statistical significance with WT was determined as above. P-values: **p = 0.015;

Supplementary Fig. 2 (continued) ns = 0.88, 0.80, 0.19, 0.97, 0.16 (from left to right). **c** Cell viability assay. A prototrophic strain and the indicated mutant derivatives (Y1724, Y1749, Y1786 & Y1802) were grown in SF-medium supplemented with 0.1 mM Met and 0.01 mM SAM before sulfur starvation. Viability was determined as described in *Methods*. Colony-forming units (CFU/mL) were counted after the indicated number of days. Data are mean of n=2 independent experiments. Statistical significance between strains was determined by two-way ANOVA with Geisser-Greenhouse correction followed Tukey's multiple comparisons tests. P-values: *p = 0.018; ns = 0.49. Source data are provided with this paper.

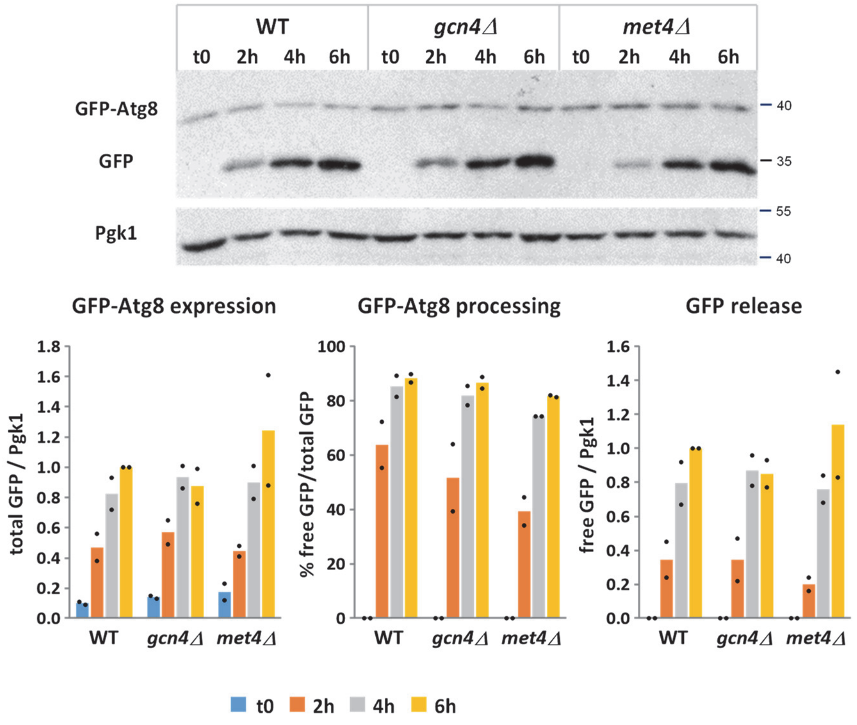

Supplementary Fig. 4. Met4 is not required to induce autophagy upon nitrogen starvation. The prototrophic strain and mutant derivatives used in Supplementary Fig. 3b (Y1727, Y1737 & Y1787) were grown to exponential phase in SF-medium supplemented with 0.1mM Met and 0.01mM SAM, and shifted into a derived medium lacking ammonium and amino acids but still containing 0.1mM Met plus 0.01mM SAM. Cells were collected at the indicated times and processed as described in *Methods*. Molecular weight are in kDa. GFP-Atg8 expression and free GFP are relative to the WT at the 6-hour time point. Data are mean of n=2 independent experiments. Source data are provided with this paper.

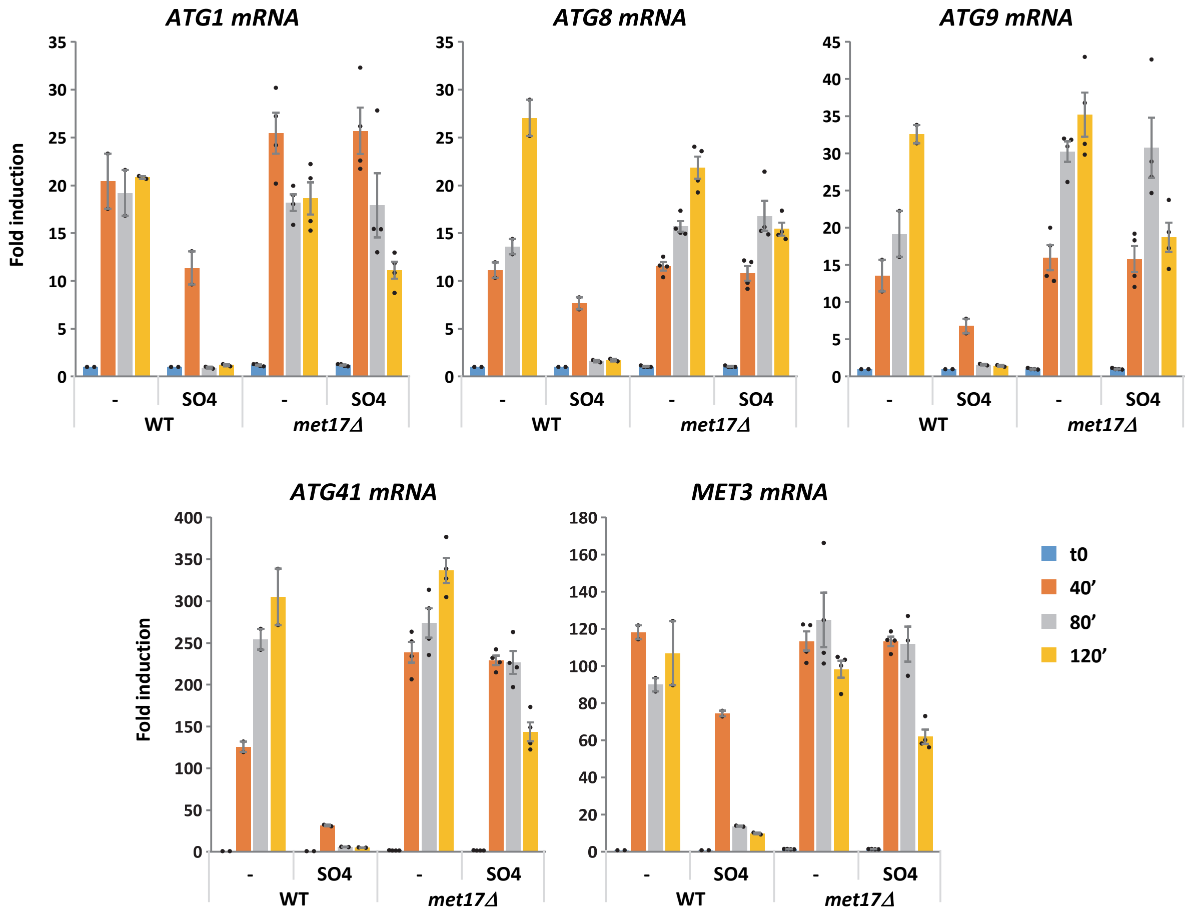

Supplementary Fig. 5. The presence of sulfate as sole source of sulfur does not prevent *ATG* gene induction in a mutant unable to assimilate sulfate into organic compounds. A WT strain and its isogenic *met17Δ* mutant (BY4742 & Y1681 or 1683) were grown in SF-medium supplemented with 0.1mM Met and shifted into SF-medium alone (-) or SF-medium supplemented with 15 mM sodium sulfate (SO4). Samples were collected at the indicated times. Transcript levels were quantified by RT-qPCR. Fold induction is relative to WT at t0. Data are mean of n=2 (WT) or n=4 independent experiments. Error bars indicate SEM. Source data are provided with this paper.

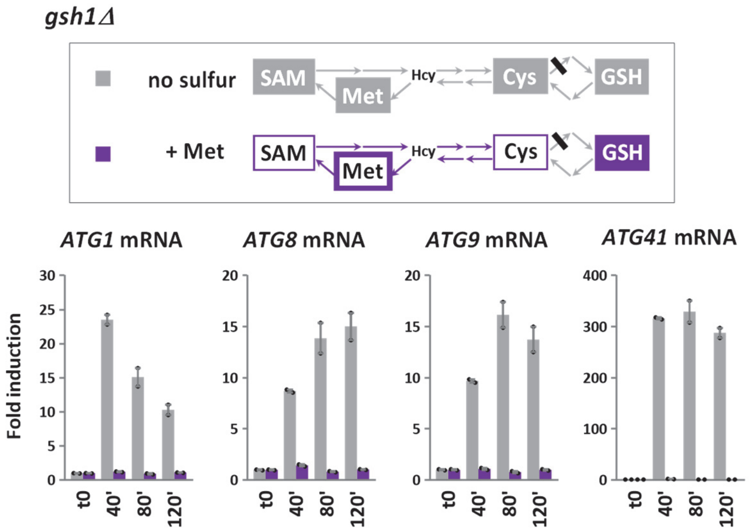

Supplementary Fig. 6. Depletion of GSH does not induce *ATG* gene expression. Simplified schematics of the sulfur-containing amino acid biosynthesis pathway indicating the steps interrupted in the *gsh1Δ* mutant (upper box). Sulfur compounds becoming depleted after transfer into the indicated starvation media are written in white on grey or purple background. Sulfur supplements are marked with a thick outline. The *gsh1Δ* strain (Y1296) was grown to in SF-medium supplemented with 0.1 mM Met and 0.005 mM GSH, and shifted to medium containing no sulfur or 0.1 mM Met. Samples were collected at the indicated times. Transcript levels (graphs) were measured by RT-qPCR. Fold induction is relative to t0. Data are mean of n=2 independent experiments. Source data are provided with this paper.

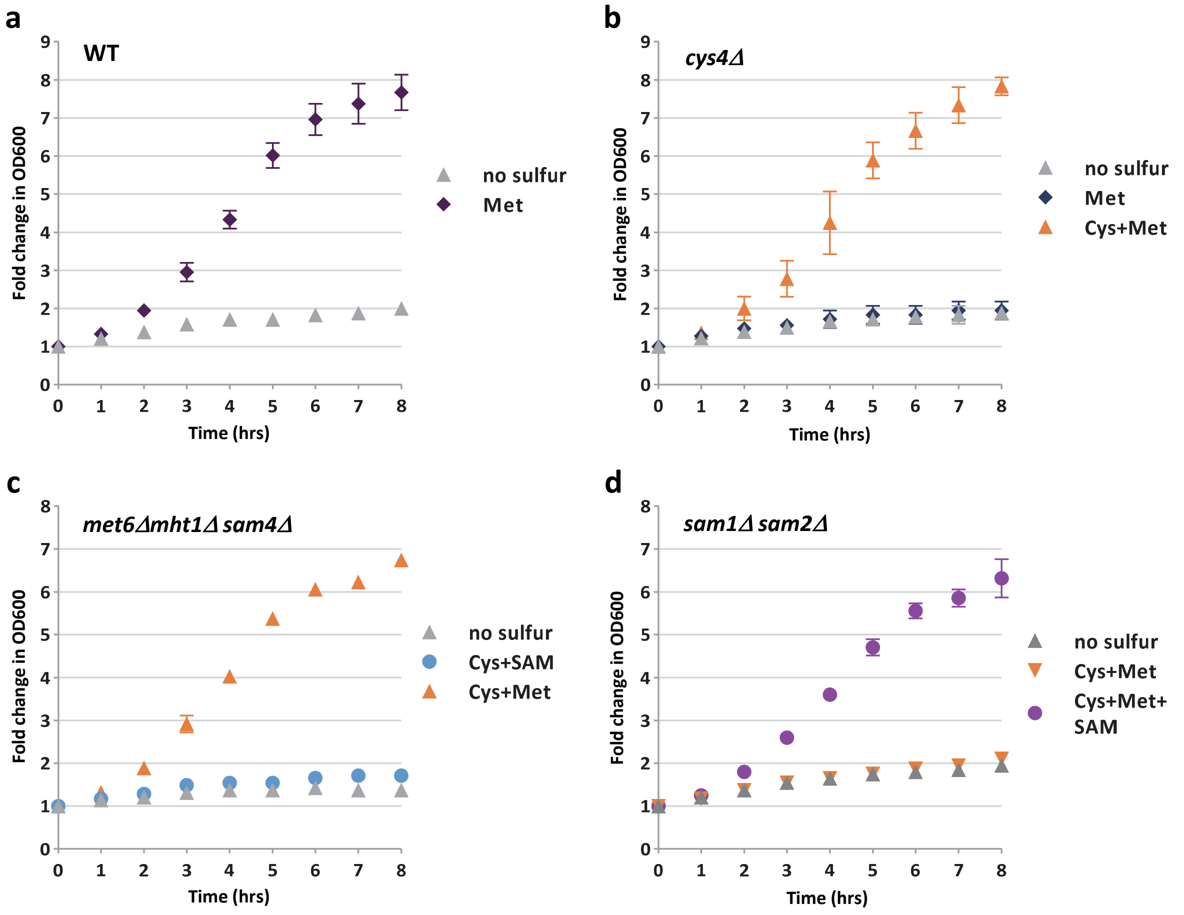

Supplementary Fig. 7. Growth profiles of the metabolic mutants under sulfur-replete or sulfur-depleted conditions. (**a**) Wild-type (BY4742), (**b**) *cys4Δ* (1677), (**c**) *met6Δ mht1Δ sam4Δ* (1533), and (**d**) *sam1Δ sam2Δ* (Y1504) cells were first grown overnight in SF-medium supplemented with either (**a**) 0.1mM Met, (**b**) 0.5mM Cys & 0.1mM Met, (**c**) 0.1mM Cys & Met, and (**d**) 0.1mM Cys, Met & SAM. At OD600 between 0.8 and 1, cultures were filtered, and cells were washed and resuspended at similar OD600 into SF-medium (no sulfur) or SF-medium supplemented with (**a**) 0.1mM Met, (**b**) 0.1mM Met or 0.1mM Met & 0.5mM Cys, (**c**) 0.1mM Cys & SAM or 0.1mM Cys & Met, and (**d**) 0.1mM Cys & Met or 0.1mM Cys, Met & SAM. Growth was monitored by measuring OD600 every hour for 8 hours. Data are mean ± SD (n=2 independent experiments). Source data are provided with this paper.

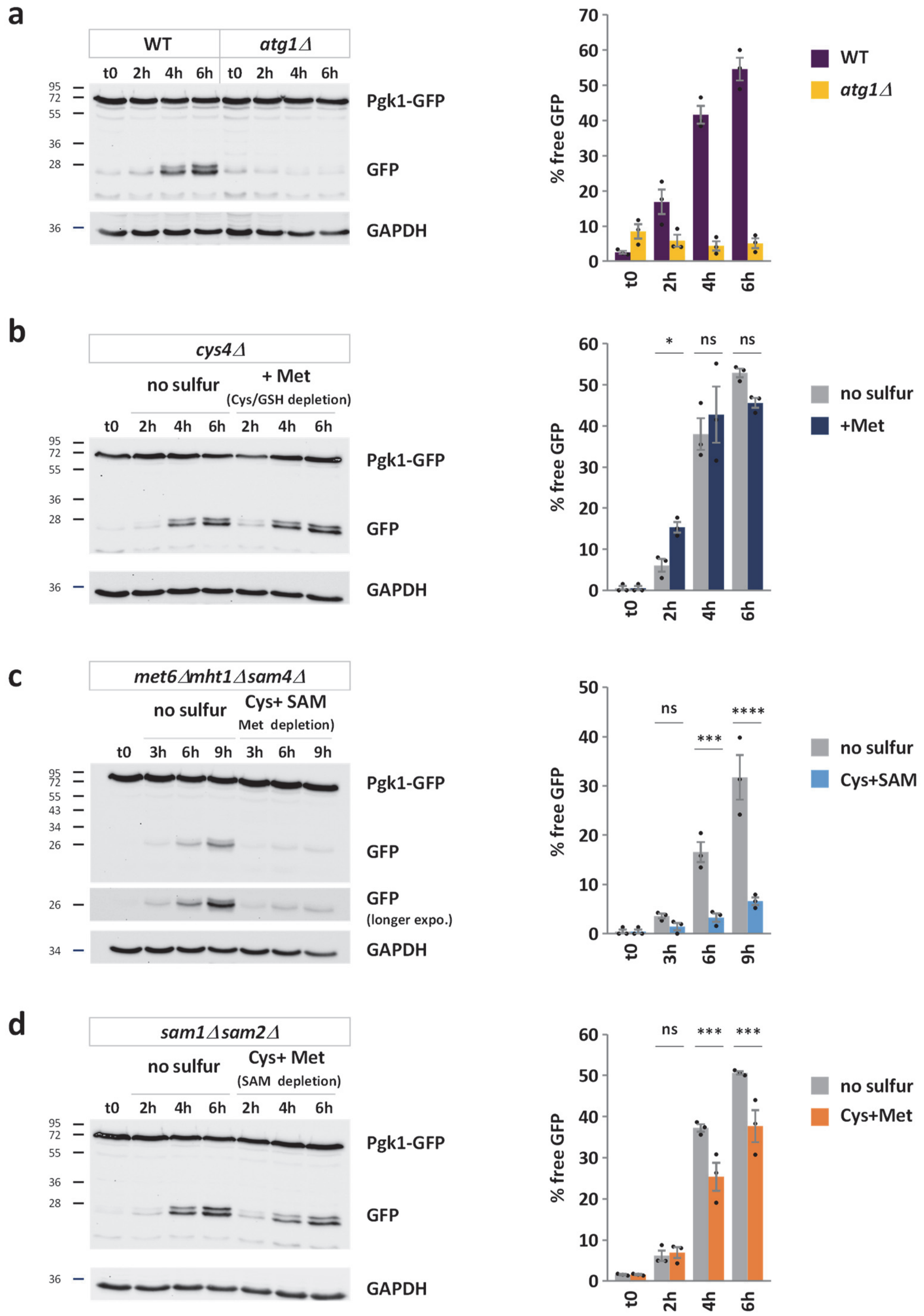

Supplementary Fig. 8. Sulfur starvation induces autophagy-dependent degradation of Pgk1-GFP. (**a**) WT and *atg1Δ* cells expressing Pgk1-GFP (Y1813 and Y1819) were grown to exponential phase in SF-medium supplemented with 0.1mM Met, collected by filtration, and transferred into SF-medium with no supplement. Cell samples were collected before at the indicated times. Protein extracts were resolved by SDS-PAGE and analyzed by Western blot using antibodies against GFP and GAPDH (loading control). Molecular weights are in kDa. Pgk1-GFP processing was quantified as the percentage of free GFP relative to GFP total amount. (**b**) *cys4*Δ (Y1834), (**c**) *met6Δ mht1Δ sam4Δ* (Y1831), and (**d**) sam1*Δ sam2Δ* (Y1866) mutant cells expressing Pgk1-GFP were grown to exponential, collected by filtration, and transferred into SF-medium with no supplement or supplemented with (**b**) 0.1mM Met, (**c**) 0.1mM Cys & SAM, and (**d**) 0.1mM Cys & Met. Cell samples were collected at the indicated times, and Pgk1-GFP processing was quantified as above. Data are mean ± SEM (n=3 independent experiments). Statistical significance between conditions was determined by multiple two-sided t-test comparisons using Holm-Sidak method. P-values: ****p < 0.0001; ***p = 0.00011, 0.00058, 0.00025; *p = 0.041, ns = 0.27, 0.097, 0.42, 0.80 (from top to bottom and left to right). Source data are provided with this paper.

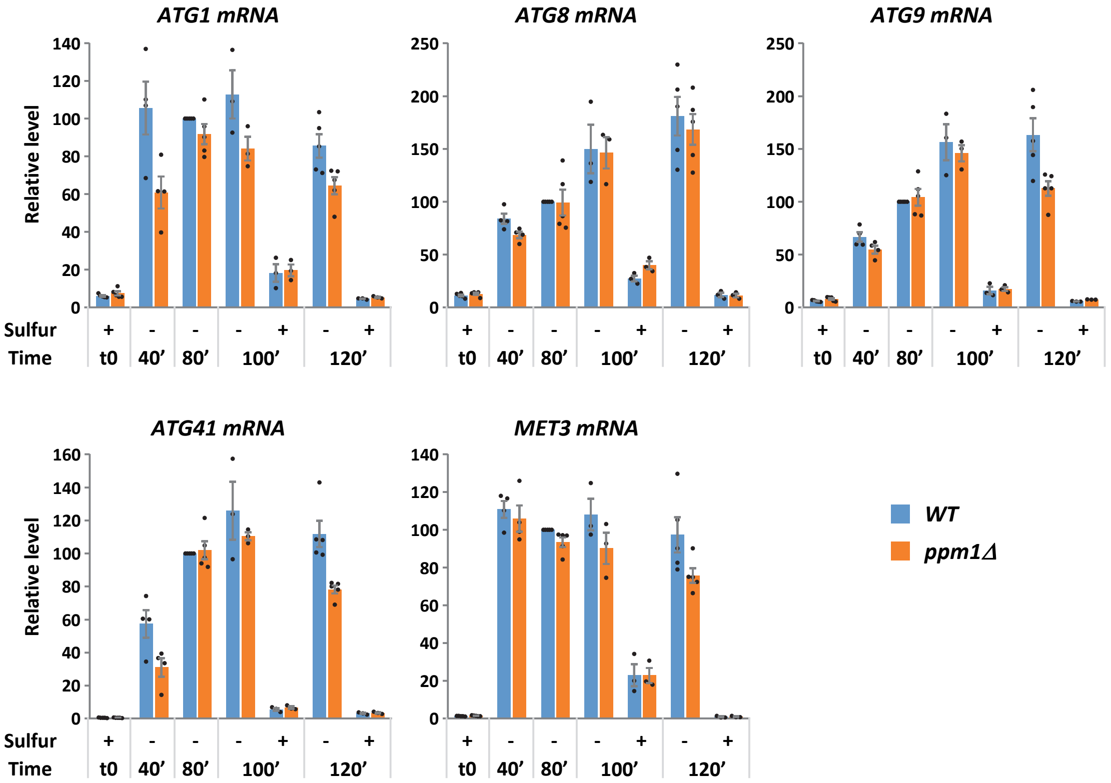

Supplementary Fig. 9. Ppm 1 is not required for *ATG* gene induction and repression in response to sulfur availability. A WT strain and its isogenic *ppm1Δ* mutant (BY4742 & Y1548) were grown to exponential phase in SF-medium supplemented with 0.1mM Met, and shifted at t0 into SF-medium without sulfur (-). After 80 min, 0.1mM Met was added back to half of the culture (+). Cell samples were collected before the shift (t0), and at the indicated time points afterwards. Transcript levels (graphs) were measured by RT-qPCR, and are relative to the WT at the 80-min time point. Data are mean ± SEM (n=3 independent experiments). Source data are provided with this paper.

Supplementary Table 1. ***Saccharomyces cerevisiae*** **strains**

Strain	Relevant genotype	Source
BY4741	*his3Δ1 leu2Δ0 met15Δ0 ura3Δ0*	KO collection
BY4742	*his3Δ1 leu2Δ0 lys2Δ0 ura3Δ0*	KO collection
Y4720	*lys2Δ0 trp1Δ63 ura3Δ0*	Fred Winston
Y614	*his3Δ1/his3Δ1 leu2Δ0/leu2Δ0 lys2Δ0/LYS2 MET15/met15Δ0 ura3Δ0/ura3Δ0 met4::kanMX4/MET4*	KO collection
Y1296	*his3Δ1 leu2Δ0 lys2Δ0 ura3Δ0 gsh1Δ::KanMX4*	KO collection
Y1397	*his3Δ1 leu2Δ0 lys2Δ0 ura3Δ0 atg1Δ::KanMX4*	KO collection
Y1406	*his3Δ1 leu2Δ0 lys2Δ0 ura3Δ0 atg9Δ::KanMX4*	KO collection
Y1407	*his3Δ1 leu2Δ0 met17Δ0 ura3Δ0 atg8::GFP-ATG8-URA3*	This study
Y1408	*his3Δ1 leu2Δ0 lys2Δ0 ura3Δ0 atg8::GFP-ATG8-URA3*	This study
Y1412/1413	*his3Δ1 leu2Δ0 ura3Δ0 atg41Δ::KanMX4 atg8::GFP-ATG8-URA3*	This study
Y1424	*his3Δ1 leu2Δ0 lys2Δ0 ura3Δ0 atg3Δ::KanMX4*	KO collection
Y1425	*his3Δ1 leu2Δ0 lys2Δ0 ura3Δ0 atg7Δ::KanMX4*	KO collection
Y1426	*his3Δ1 leu2Δ0 lys2Δ0 ura3Δ0 atg8Δ::KanMX4*	KO collection
Y1437	*his3Δ1 leu2Δ0 lys2Δ0 ura3Δ0 gcn4Δ::KanMX4*	KO collection
Y1439	*his3Δ1 leu2Δ0 lys2Δ0 ura3Δ0 met4Δ::KanMX4*	This study
Y1446	*his3Δ1 leu2Δ0 lys2Δ0 ura3Δ0 atg41Δ::KanMX4*	This study
Y1489	*his3Δ1 leu2Δ0 lys2Δ0 ura3Δ0 met6Δ::KanMX4*	KO collection
Y1490	*his3Δ1 leu2Δ0 lys2Δ0 ura3Δ0 sam1Δ::KanMX4*	KO collection
Y1491	*his3Δ1 leu2Δ0 lys2Δ0 ura3Δ0 sam2Δ::KanMX4*	KO collection
Y1504	*his3Δ1 leu2Δ0 lys2Δ0 ura3Δ0 sam1Δ::KanMX4 sam2Δ::KanMX4*	This study
Y1506	*his3Δ1 leu2Δ0 lys2Δ0 ura3Δ0 sam1Δ::KanMX4 sam2Δ::KanMX4 atg8::GFP-ATG8-URA3*	This study
Y1511	*his3Δ1 leu2Δ0 lys2Δ0 ura3Δ0 sam4Δ::KanMX4*	This study
Y1526	*his3Δ1 leu2Δ0 lys2Δ0 trp1Δ63 ura3Δ0 MET4::9myc-TRP1*	This study
Y1533/1534	*his3Δ1 leu2Δ0 lys2Δ0 ura3Δ0 met6Δ::KanMX4 mht1Δ::S.kluy.HIS3 sam4Δ::KanMX4*	This study
Y1535/1536	*his3Δ1 leu2Δ0 lys2Δ0 ura3Δ0 met6Δ::KanMX4 mht1Δ::S.kluy.HIS3 sam4Δ::KanMX4 atg8::GFP-ATG8-URA3*	This study
Y1539	*ura3Δ0/uraΔ3 his3Δ1/his3Δ1 leu2Δ0/leu2Δ LYS2/lys2Δ0 MET15/met15Δ0 trp1D63/trp1D63*	This study
Y1548	*his3Δ1 leu2Δ0 lys2Δ0 ura3Δ0 ppm1Δ::KanMX4*	KO collection
Y1558	*his3Δ1 leu2Δ0 lys2Δ0 ura3Δ0 cys4Δ::KanMX4 atg8::GFP-ATG8-URA3*	This study
Y1559	*his3Δ1 leu2Δ0 lys2Δ0 ura3Δ0 cys4Δ::KanMX4*	KO collection
Y1571	*his3Δ1 leu2Δ0 lys2Δ0 ura3Δ0 gcn4Δ::KanMX4 met4Δ::KanMX4*	This study
Y1578	*his3Δ1 leu2Δ0 lys2Δ0 trp1Δ63 ura3Δ0 GCN4::9myc-TRP1*	This study
Y1583	*his3Δ1 leu2Δ0 lys2Δ0 ura3Δ0 met4Δ::His3MX6 atg8::GFP-ATG8-URA3*	This study
Y1601	*his3Δ1 leu2Δ0 lys2Δ0 ura3Δ0 met4Δ::His3MX6 sam1Δ::KanMX4 sam2Δ::KanMX4*	This study
Y1608	*his3Δ1 leu2Δ0 lys2Δ0 ura3Δ0 gcn4Δ::KanMX4 met4Δ::His3MX6 atg8::GFP-ATG8-URA3*	This study
Y1611	*his3Δ1 leu2Δ0 lys2Δ0 ura3Δ0 gcn4Δ::KanMX4 atg8::GFP-ATG8-URA3*	This study

Supplementary Table 1. Continued

Strain	Relevant genotype	Source
Y1628/1629	*his3Δ1 leu2Δ0 lys2Δ0 ura3Δ0 pho8::K.l.URA3-ADH1p-pho8Δ60*	This study
Y1638	*his3Δ1 leu2Δ0 lys2Δ0 ura3Δ0 atg41Δ::KanMX4 pho8::K.l.URA3-ADH1p-pho8Δ60*	This study
Y1643	*his3Δ1 leu2Δ0 lys2Δ0 ura3Δ0 atg1Δ::KanMX4 pho8::K.l.URA3-ADH1p-pho8Δ60*	This study
Y1660	*his3Δ1 leu2Δ0 lys2Δ0 ura3Δ0 cys4Δ::LEU2 met6Δ::KanMX4 mht1Δ::S.kluy.HIS3sam4Δ::KanMX4*	This study
Y1677	*his3Δ1 leu2Δ0 lys2Δ0 ura3Δ0 cys4Δ::KanMX4*	This study
Y1681/1683	*his3Δ1 leu2Δ0 lys2Δ0 ura3Δ0 met17Δ::KanMX4*	This study
Y1724/1725	no auxotrophy	This study
Y1718/1720	*his3Δ1 leu2Δ0 lys2Δ0 ura3Δ0 gcn4Δ::KanMX4 pho8::K.l.URA3-ADH1p-pho8Δ60*	This study
Y1722/1765	*his3Δ1 leu2Δ0 lys2Δ0 ura3Δ0 met4Δ::KanMX4 pho8::K.l.URA3-ADH1p-pho8Δ60*	This study
Y1723/1748	*his3Δ1 leu2Δ0 lys2Δ0 ura3Δ0 gcn4Δ::KanMX4 met4Δ::KanMX4 pho8::K.l.URA3-ADH1p-pho8Δ60*	This study
Y1727	*ura3Δ0 atg8::GFP-ATG8-URA3*	This study
Y1746	*gcn4Δ::KanMX4*	This study
Y1786	*trp1Δ63 met4Δ::TRP1*	This study
Y1737	*ura3Δ0 gcn4Δ::KanMX4 atg8::GFP-ATG8-URA3*	This study
Y1787	*trp1Δ63 ura3Δ0 met4Δ::TRP1 atg8::GFP-ATG8-URA3*	This study
Y1749	*atg1Δ::KanMX4*	This study
Y1802	*trp1Δ63 atg1Δ::KanMX4 met4Δ::TRP1*	This study
Y1813	*his3Δ1 leu2Δ0 lys2Δ0 ura3Δ0 PGK1::GFP-hphNT1*	This study
Y1819	*his3Δ1 leu2Δ0 lys2Δ0 ura3Δ0 atg1Δ::KanMX4 PGK1::GFP-hphNT1*	This study
Y1831	*his3Δ1 leu2Δ0 lys2Δ0 ura3Δ0 met6Δ::KanMX4 mht1Δ::S.kluy.HIS3 sam4Δ::KanMX4 PGK1::GFP-hphNT1*	This study
Y1834	*his3Δ1 leu2Δ0 lys2Δ0 ura3Δ0 cys4Δ::KanMX4 PGK1::GFP-hphNT1*	This study
Y1866	*his3Δ1 leu2Δ0 lys2Δ0 ura3Δ0 sam1Δ::KanMX4 sam2Δ::KanMX4 PGK1::GFP-hphNT1*	This study

Supplementary Table 2. Sulfur-free (SF) medium, nucleotides and amino acids

Component		Amount (mg/L)	Concentration
Carbone source	Glucose		2%
Nitrogen source	Ammonium chloride	800	15 mM
Salts	Potassium phosphate monobasic	900	6.6 mM
	Potassium phosphate dibasic	120	0.5 mM
	Magnesium chloride	400	2 mM
	Sodium chloride	100	1.7 mM
	Calcium chloride	100	0.7 mM
Vitamins	D-Biotin	0.002	0.08 μM
	Calcium pantothenate	0.1	4 μM
	Folic acid	0.002	0.0045 μM
	Myo-Inositol	10	55 μM
	Nicotinic acid	0.4	2.9 μM
	p-Aminobenzoic acid	0.2	1.5 μM
	Pyridoxine hydrochloride	2	10 μM
	Riboflavin	0.2	0.53 μM
	Thiamine hydrochloride	2	6 μM
Trace elements	Boric acid	0.5	8 μM
	Copper chloride	0.02	0.2 μM
	Potassium iodide	0.1	0.6 μM
	Ferric chloride	0.03	0.18 μM
	Manganese chloride	0.3	2.4 μM
	Sodium molybdate	0.2	0.8 μM
	Zinc chloride	0.2	1.4 μM
Nucleotides	Adenine	20	148 μM
	Uracil	20	178 μM
Amino acids	L-Alanine	40	449 μM
	L-Arginine	40	230 μM
	L-Asparagine	40	303 μM
	L-Aspartate	100	751 μM
	L-Glutamine	40	279 μM
	L-Glutamate	100	680 μM
	L-Glycine	40	553 μM
	L-Histidine	20	129 μM
	L-Isoleucine	40	305 μM
	L-Leucine	60	457 μM
	L-Lysine-HCl	40	274 μM
	L-Phenylalanine	50	303 μM
	L-Proline	40	347 μM
	L-Serine	200	1.9 mM
	L-Threonine	200	1.6 mM
	L-Tryptophan	20	98 μM
	L-Tyrosine	40	221 μM
	L-Valine	150	1.2 mM

Supplementary Table 3. Sequence of primers used in the RT-qPCR experiments

Gene	Oligo #	Strand	Position (to ATG)	Sequence 5'->3'
ATG1	2077	Fwd	+41	ACCATAATCTAATGGCAAGTGCAGG
	2078	Rev	+176	GCCCTTGATACTTCCTTTATGGCT
ATG8	2079	Fwd	+62	TCGGAGAGGATTGCTGACAGGT
	2080	Rev	+184	AAACAAATTGCCCTACGGTAAGGTC
ATG9	2081	Fwd	+54	ATCGCGAATATTTGGTTTACAATCG
	2082	Rev	+198	ATTCTCATCTACATCTTCGCGGCT
ATG14	2243	Fwd	+24	TAGAGCGCATGTAGTGTACTGT
	2244	Rev	+229	TGGCCTCGTTTAATATTTGTTCGA
ATG29	2318	Fwd	+229	GCTGAACACTTAGAGCTTTTACAACT
	2319	Rev	+386	AGTGGGCTTGCGGATACATTTA
ATG39	2253	Fwd	+149	ATGCAGATGTCCTATCGAACACTA
	2254	Rev	+390	ATCCGATAGTGTGCTAGAATTCCT
ATG41	2075	Fwd	+7	TCTGTGGAATCTTCCCCCATCT
	2076	Rev	+145	GGTAAGATTGAGAAAAGTCATCGGC
MEP2	2287	Fwd	+129	GATTATGGTCCCAGGTATCGGTTT
	2288	Rev	+324	ACGAAACCCAAAGAATTCCAAGGT
MET3	2130	Fwd	+168	GACTGGGTTTTTGAACGAAAACG
	2131	Rev	+347	TTGGCTTGTAAACATCCTGGACA
CCW12	2100	Fwd	+230	AAGCCCCAAAGAACGGTACTTCTA
	2101	Rev	+353	AAAGCCTTAGCAGCAGCACC
25S rDNA	394	Fwd	+624	GGTTATATGCCGCCCGTCTTGA
	395	Rev	+800	CCCAACAGCTATGCTCTTACTC

Supplementary Table 4. Sequence of primers used in the ChIP experiments

Gene (orf size)	Oligo#	Strand	Position (to ATG)	Sequence 5'->3'
ATG1_A	2186	Fwd	-404	TCGATGGAATTCTAAGCGTCATTT
	2187	Rev	-182	ATAGTACTGTTCTCTGTTTCCCCA
ATG1_B	2237	Fwd	-843	CTATTGTATTCACCCTACACAGCC
	2238	Rev	-656	TTTTCCTTACATTACCGCCCAATC
ATG8	2239	Fwd	-222	TAATGCTAACTGTCTCCACCGATA
	2240	Rev	-51	TTCTCTTATCAATCCCCTCCTCAA
ATG9	2241	Fwd	-235	TGCTTAGATTCCCATTCAAAAGGT
	2242	Rev	-67	ACTCATTACTTCACGGTGGTTATG
ATG41_A	2182	Fwd	-505	ATATTCTCCATCTGCGTTGTTTCA
	2183	Rev	-276	ATGTGGTGTGCAATGTGTCATCTT
ATG41_B	2184	Fwd	-936	AAGACCGAGTGCTATTTTCCATGG
	2185	Rev	-687	TCTAATGGAGATGAGTGAGTGGGC
IME2	328	Fwd	+1173	ATCCCAAGTAGACGCAAGAGGCAAT
	329	Rev	+1377	TTCTTGATTTAATGTTGGTGAGCACA
MET16	118	Fwd	-227	CATACTGTTCTTTATTCCGTCGTT
	119	Rev	-19	TTGATACCTTTGCTGGCCTTAGTT

Supplementary Table 5. DESeq2[1] results for the 36 Autophagy-related (*ATG*) genes

Name	baseMean	padj	log2FoldChange					
			40'/t0	SE	80'/t0	SE	120'/t0	SE
ATG1	2429	0.0E+00	2.70	0.07	2.94	0.07	2.93	0.07
ATG2	1089	1.3E-42	0.55	0.07	0.51	0.07	0.94	0.07
ATG3	265	3.7E-32	0.70	0.13	1.18	0.13	1.44	0.13
ATG4	341	1.1E-63	1.24	0.13	1.62	0.13	1.99	0.12
ATG5	753	8.7E-82	1.64	0.09	1.40	0.09	1.56	0.09
ATG6/VPS30	383	3.1E-23	0.31	0.11	0.62	0.11	1.00	0.11
ATG7	882	1.9E-180	1.19	0.09	1.67	0.09	2.31	0.09
ATG8	451	2.3E-191	2.21	0.14	2.54	0.14	3.47	0.14
ATG9	2005	0.0E+00	2.36	0.08	2.91	0.08	3.62	0.08
ATG10	25	9.0E-01	0.09	0.36	0.01	0.36	-0.17	0.37
ATG11	834	3.0E-43	0.72	0.08	0.74	0.08	1.14	0.08
ATG12	163	9.3E-14	0.97	0.16	1.10	0.16	1.18	0.16
ATG13	379	8.2E-07	0.65	0.14	0.69	0.14	0.66	0.14
ATG14	239	5.5E-89	1.87	0.18	2.62	0.18	3.08	0.18
ATG15	1237	4.3E-114	1.17	0.07	1.07	0.07	1.66	0.07
ATG16	105	2.4E-25	1.60	0.22	1.83	0.22	2.21	0.22
ATG17	159	3.7E-11	0.62	0.16	1.03	0.16	0.97	0.16
ATG18	708	3.9E-07	0.41	0.08	0.38	0.08	0.33	0.08
ATG19	1649	2.6E-256	1.42	0.07	1.72	0.07	2.16	0.07
ATG20	1377	5.3E-201	1.53	0.08	1.74	0.08	2.37	0.08
ATG21	668	1.7E-56	0.88	0.09	1.15	0.09	1.47	0.09
ATG22	1587	9.3E-181	0.99	0.07	1.40	0.07	1.92	0.07
ATG23	237	4.3E-12	0.26	0.13	0.72	0.13	0.78	0.13
ATG24/SNX4	643	7.5E-48	0.61	0.09	0.86	0.09	1.27	0.09
ATG27	641	9.1E-55	-0.83	0.08	-1.09	0.08	-1.00	0.08
ATG29	435	2.8E-150	2.18	0.14	2.54	0.14	3.19	0.14
ATG31	316	5.5E-45	0.77	0.12	1.19	0.12	1.59	0.12
ATG32	742	4.7E-189	1.04	0.10	1.72	0.09	2.42	0.09
ATG33	763	5.1E-91	0.97	0.09	1.19	0.09	1.82	0.09
ATG34	480	5.2E-162	1.37	0.12	1.99	0.12	2.75	0.12
ATG36	558	1.9E-140	1.44	0.11	1.73	0.11	2.50	0.11
ATG38	140	6.8E-02	0.17	0.16	0.41	0.16	0.25	0.16
ATG39	286	1.5E-217	2.65	0.26	3.64	0.25	5.14	0.25
ATG40	531	7.7E-98	1.23	0.11	1.39	0.11	2.13	0.11
ATG41	18809	0.0E+00	5.00	0.08	6.22	0.08	6.61	0.08
ATG42	4971	0.0E+00	1.41	0.05	1.43	0.05	2.13	0.05

Supplementary Table 6. DESeq2[1] results for the 44 sulfur amino acid biosynthesis genes

pathway	Name	baseMean	padj	log2FoldChange					
				40'/t0	SE	80'/t0	SE	120'/t0	SE
sulfate assimilation	HOM2	2246	7.6E-22	-0.09	0.16	-0.63	0.16	-1.57	0.16
	HOM3	16931	0.0E+00	2.19	0.05	2.62	0.05	2.29	0.05
	HOM6	22233	2.5E-137	0.67	0.05	1.01	0.05	1.07	0.05
	MET1	13686	0.0E+00	5.92	0.07	5.76	0.07	5.48	0.07
	MET2	6676	0.0E+00	3.85	0.06	4.34	0.06	4.20	0.06
	MET3	36264	0.0E+00	5.13	0.05	4.89	0.05	4.97	0.05
	MET5	60301	0.0E+00	3.86	0.04	3.60	0.04	3.30	0.04
	MET8	1494	0.0E+00	4.48	0.12	4.30	0.12	3.65	0.12
	MET10	109403	0.0E+00	4.65	0.04	4.70	0.04	4.95	0.04
	MET14	12592	0.0E+00	4.11	0.05	3.99	0.05	3.97	0.05
	MET16	5065	0.0E+00	5.08	0.09	4.96	0.09	4.78	0.09
	MET17	130526	0.0E+00	2.93	0.04	2.97	0.04	3.35	0.04
	MET22	7721	0.0E+00	3.14	0.05	2.71	0.05	2.83	0.05
trans-sulfuration	CYS3	44581	0.0E+00	2.48	0.05	2.48	0.05	2.71	0.05
	CYS4	24837	0.0E+00	1.45	0.04	1.23	0.04	1.18	0.04
	STR2	1143	7.0E-190	2.21	0.08	1.97	0.08	1.57	0.08
	STR3	15387	0.0E+00	6.12	0.07	6.34	0.07	6.16	0.07
GSH cycle	DUG1	6870	4.9E-28	-0.08	0.04	0.03	0.04	0.35	0.04
	DUG2	2357	1.7E-196	1.53	0.06	1.49	0.06	1.50	0.06
	DUG3	2754	0.0E+00	3.11	0.07	3.38	0.07	3.71	0.07
	ECM38	833	8.5E-80	1.24	0.08	1.00	0.08	1.42	0.08
	GSH1	9374	0.0E+00	3.15	0.05	3.04	0.05	3.53	0.05
	GSH2	1273	4.3E-11	-0.32	0.06	-0.41	0.06	-0.12	0.06
methyl cycle	MET6	167981	8.5E-303	1.48	0.04	1.08	0.04	1.30	0.04
	MHT1	3998	0.0E+00	4.28	0.09	4.53	0.09	4.91	0.09
	SAH1	14563	0.0E+00	-2.00	0.05	-2.47	0.05	-2.77	0.05
	SAM1	18821	1.5E-65	0.46	0.04	-0.17	0.04	-0.10	0.04
	SAM2	29166	0.0E+00	2.08	0.04	1.89	0.04	2.22	0.04
	SAM4	1392	1.7E-298	-0.93	0.06	-1.65	0.06	-2.28	0.07
folate transformation	MET7	821	4.0E-17	-0.01	0.07	-0.23	0.07	-0.55	0.07
	MET12	1301	1.2E-22	0.50	0.06	0.36	0.06	0.62	0.06
	MET13	6556	1.3E-237	1.73	0.05	1.06	0.05	1.01	0.05
polyamine biosynthesis	SPE2	458	8.8E-77	-1.03	0.09	-1.14	0.09	-1.71	0.10
	SPE3	2669	0.0E+00	-1.81	0.06	-2.20	0.06	-2.36	0.06
	SPE4	333	3.9E-51	-0.72	0.09	-1.12	0.10	-1.48	0.10
MTA cycle	ADI1	1656	0.0E+00	2.81	0.09	3.15	0.09	3.35	0.09
	ARO8	7860	7.3E-187	-0.08	0.04	-0.72	0.04	-1.16	0.05
	ARO9	6457	0.0E+00	3.99	0.08	4.17	0.08	5.64	0.08
	BAT1	5518	0.0E+00	-2.29	0.04	-3.53	0.05	-4.90	0.06
	BAT2	2046	2.0E-18	0.44	0.06	0.04	0.06	0.01	0.06
	MDE1	637	1.9E-40	-0.73	0.08	-0.90	0.08	-0.96	0.08
	MEU1	656	1.6E-253	-1.87	0.07	-1.95	0.08	-2.10	0.08
	MRI1	1048	2.0E-171	-1.11	0.06	-1.46	0.06	-1.56	0.07
	UTR4	768	1.1E-04	-0.10	0.08	-0.05	0.08	0.24	0.08

Supplementary Table 7. Position and sequence of possible binding sites for Cbf1, Met31/32 and Gcn4

	Cbf1 binding site TCAC(G/A)TG[2]			Met31 and Met32 binding site (AAA)CTGTGGC[2]			Gcn4 binding site TGA(C/G)TCA[3]		
	Site	Position	Strand	Site	Position	Strand	Site	Position	Strand
ATG1	none			gAAaTGTGGC	-264	Lower	TGACTaA	-778	Upper
				tAAgTGTGGa	-756	Lower	TGACTaA	-856	Lower
				AcctTGTGGC	-996	Lower			
ATG8	gCACATG	-171	Upper	gAAgTGTGGt	-531	Upper	TGACTCt	-231	Lower
ATG9	none			AAAgTGTGGC	-116	Lower	none		
				AAAaTGTGGt	-421	Lower			
ATG41	TCACGTG	-368	Lower	AAgCTGTGGC	-432	Upper	TGACaCA	-295	Upper
	TCACGTG	-770	Upper	tgACTGTGGC	-966	Lower			

Supplementary References

1. Love, M. I., Huber, W. & Anders, S. Moderated estimation of fold change and dispersion for RNA-seq data with DESeq2. *Genome Biol.* **15**, 550 (2014).
2. Cormier, L., Barbey, R. & Kuras, L. Transcriptional plasticity through differential assembly of a multiprotein activation complex. *Nucleic Acids Res.* **38**, 4998–5014 (2010).
3. Gordân, R. *et al.* Curated collection of yeast transcription factor DNA binding specificity data reveals novel structural and gene regulatory insights. *Genome Biol.* **12**, R125 (2011).
